# Supplementary material for: Two Annulated Azaheterocyclic Cores Readily Available from a Single Tetrahydroisoquinolonic Castagnoli–Cushman Precursor
Source: Molecules. 2020 Apr 28;25(9):2049. doi: 10.3390/molecules25092049 (PMC7248902; doi:10.3390/molecules25092049)
Supplement: Supplementary file 1 [file molecules-25-02049-s001.pdf]

Supporting Information for

Two annulated azaheterocyclic cores readily available from a single tetrahydroisoquinolonic Castagnoli-Cushman precursor

Elizaveta Karchuganova,[a] Olga Bakulina,[a] Dmitry Dar'in[a] and Mikhail Krasavin*[a]

[a]*Saint Petersburg State University, Saint Petersburg 199034, Russian Federation*

* Corresponding Author; phone: + 7 931 3617872, fax: +7 812 428 6939.

E-mail: m.krasavin@spbu.ru; https://krasavin-group.org/

Table of contents

1. Table S1. Reaction conditions screening for the reduction of compound **5** **S2**

2. Crystallographic data for compound **7** **S2**

3. Copies of NMR spectra for compounds **2,4,5,7-11** **S4**

4. References **S12**

1. Table S1. Reaction conditions screening for the reduction of compound **5**

**Entry**	**Reagents and conditions**	**Result** (according to NMR)
1	$Na_2S_2O_4$, (4 equiv.), DME-H_2O (1:1), reflux, 16h	Decomposition of substrate
2	$SnCl_2$ (5 equiv.), EtOH, reflux, 16h	Decomposition of substrate
3	H_2 (1 atm), THF, 10% wt Pd/C, r.t., 16h	Full conversion. Two products (3:1) - compound **4** + corresponding NH-lactam (overreduction)
4	HCO_2H (50 equiv.), MeOH-THF (1:1), 10% wt Pd/C, reflux, 16h	Full conversion. Compound **4** + two unknown by-products (2:1:1)
5	HCO_2NH_4 (10 equiv.), MeOH, 10% wt Pd/C, reflux, 16h	Compound **4** was isolated in 96% yield
6	Na_2S (6 equiv.), dioxane-water (1:1), 70 °C, 16h	Compound **7** was isolated in 91% yield

2. Crystallographic data

X-ray single crystal analysis was performed on Agilent Technologies «Supernova» diffractometer with monochromated Cu Kα radiation. The temperature was kept at 293 K during data collection. Using Olex2[1], the structure was solved with the SHELXT[2] structure solution program using Intrinsic Phasing and refined with the SHELXL[3] refinement package using Least Squares minimization. *CCDC 1996076* contains the supplementary crystallographic data for this paper. These data can be obtained free of charge from The Cambridge Crystallographic Data Centre via http://www.ccdc.cam.ac.uk.

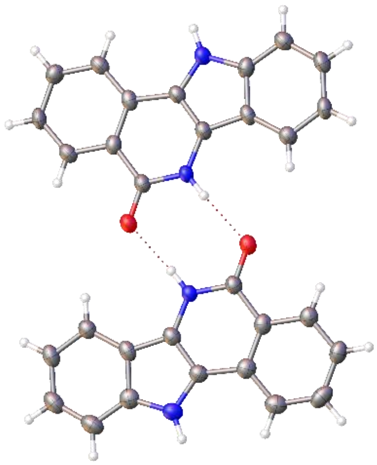

Figure S1. Crystal structure of compound **7**

Table S2. Crystal data and structure refinement for compound **7**

Empirical formula	$C_{30}H_{20}N_4O_2$
Formula weight	468.50
Temperature/K	293(2)
Crystal system	monoclinic
Space group	$P2_1/c$
a/Å	11.0070(3)
b/Å	15.9054(4)

c/Å	12.3765(4)
α/°	90
β/°	95.846(3)
γ/°	90
Volume/Å^3	2155.49(11)
Z	4
ρ_{calc}g/cm^3	1.444
μ/mm^{-1}	0.744
F(000)	976.0
Radiation	CuKα (λ = 1.54184)
2Θ range for data collection/°	8.074 to 141.34
Index ranges	-13 ≤ h ≤ 13, -19 ≤ k ≤ 19, -13 ≤ l ≤ 15
Reflections collected	23901
Independent reflections	4127 [R_{int} = 0.0518, R_{sigma} = 0.0297]
Data/restraints/parameters	4127/0/325
Goodness-of-fit on F^2	1.054
Final R indexes [I>=2σ (I)]	R_1 = 0.0686, wR_2 = 0.1930
Final R indexes [all data]	R_1 = 0.0752, wR_2 = 0.1995
Largest diff. peak/hole / e Å^{-3}	0.81/-0.33

3. Copies of NMR spectra for compounds 2,4,5,7-11

^{1}H- and ^{13}C-spectra of compound 5

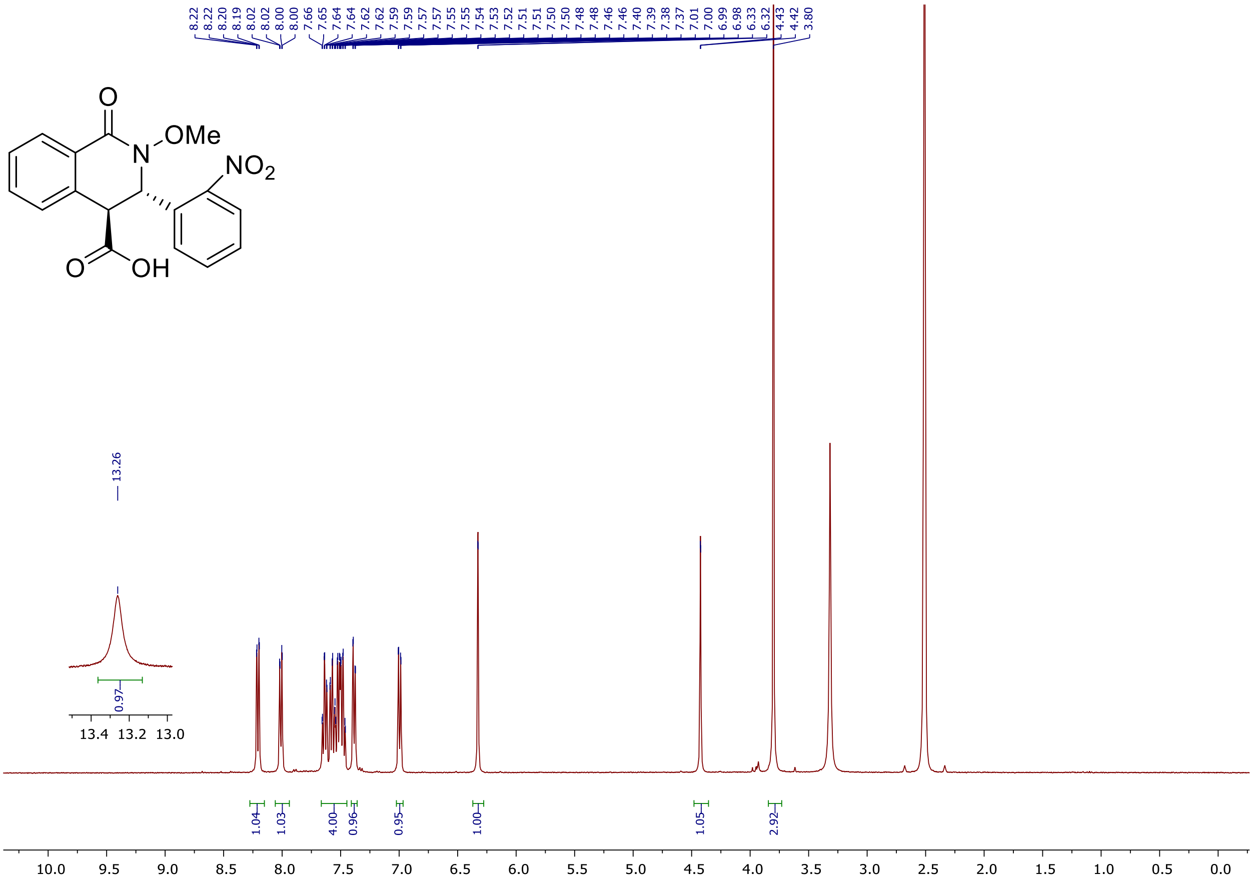

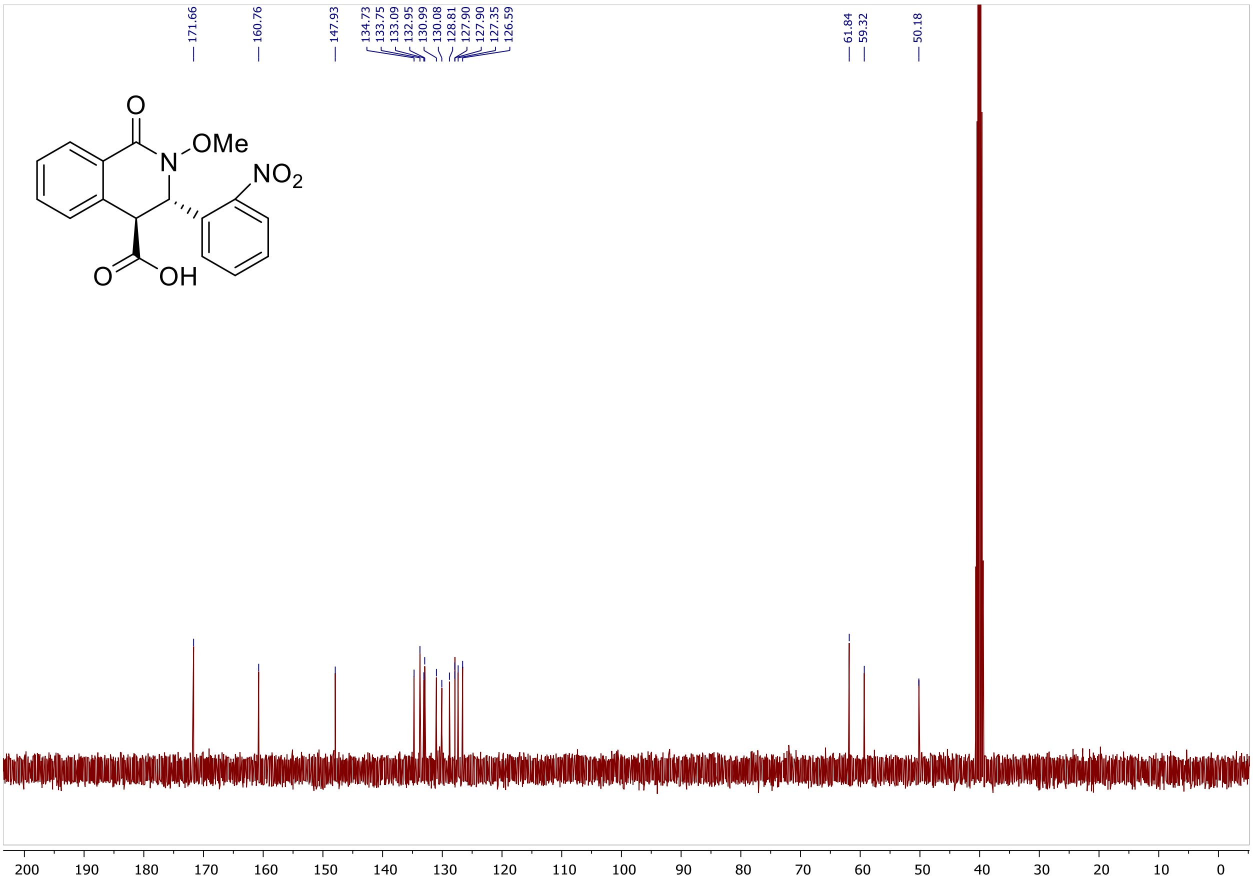

^{1}H- and ^{13}C-spectra of compound 7

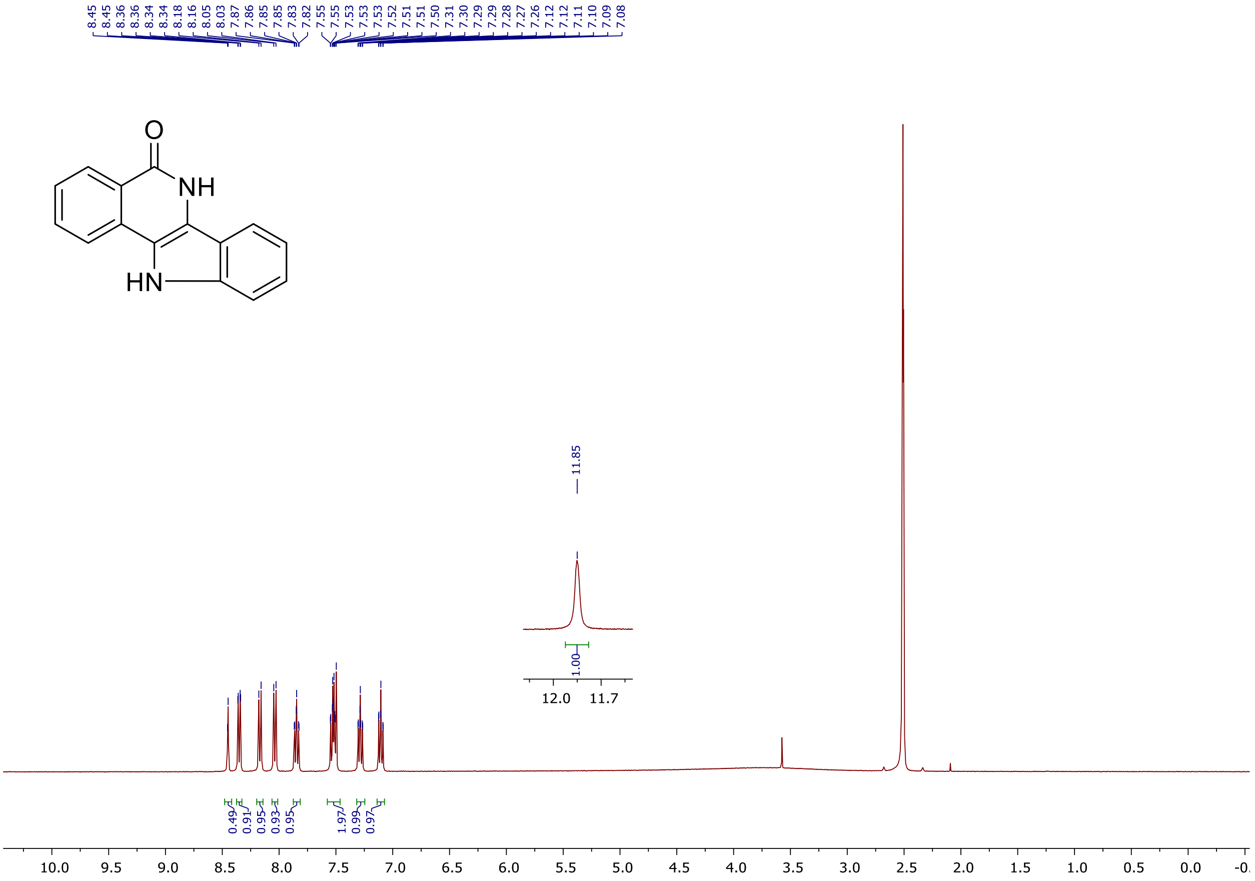

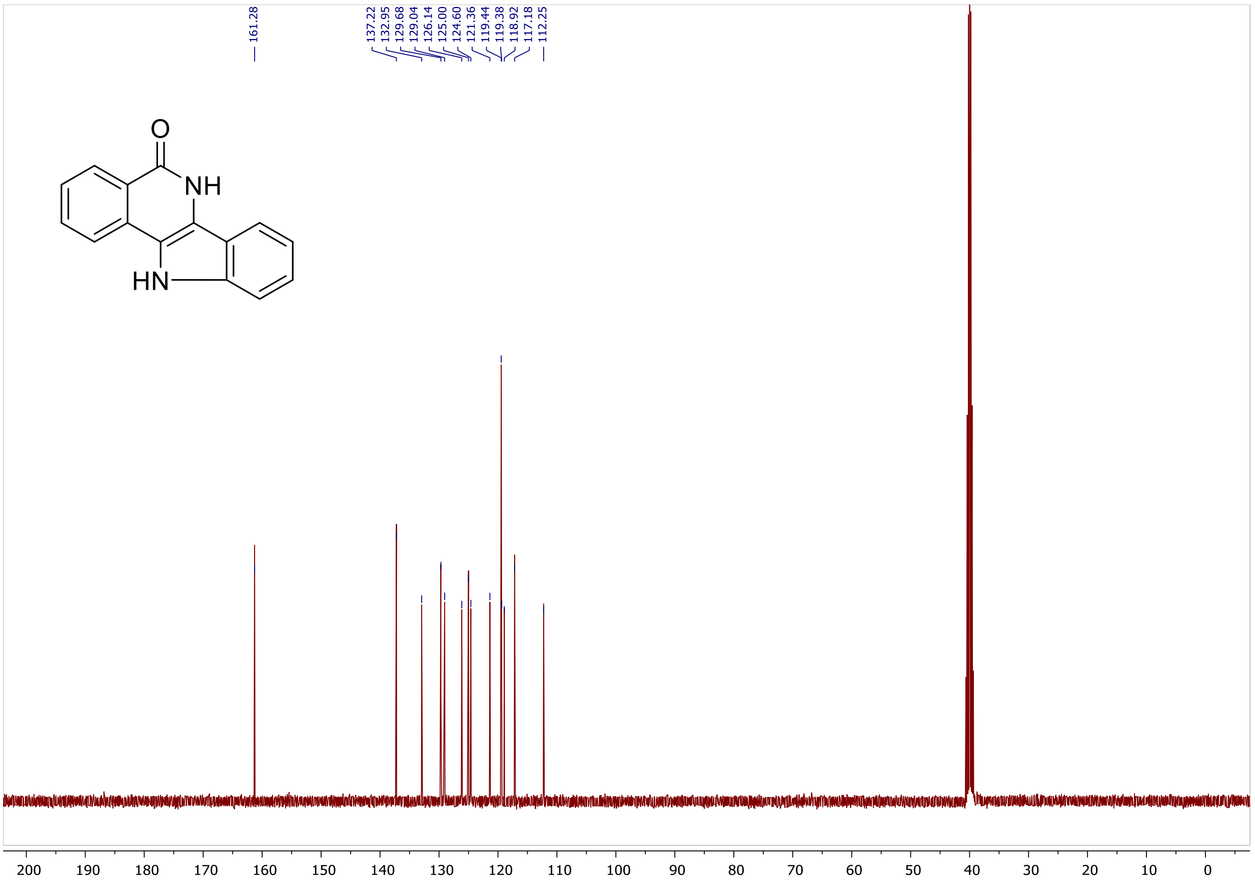

^{1}H- and ^{13}C-spectra of compound 4

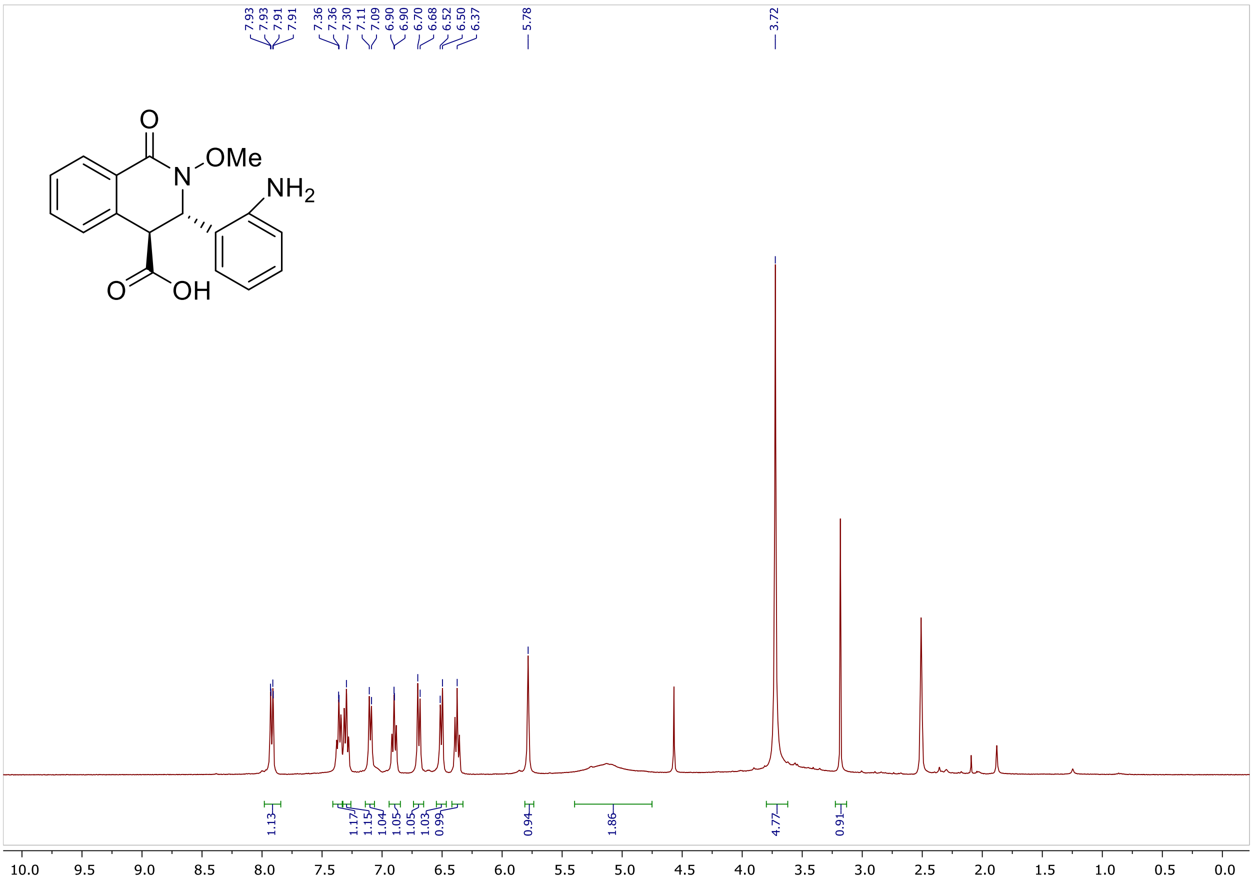

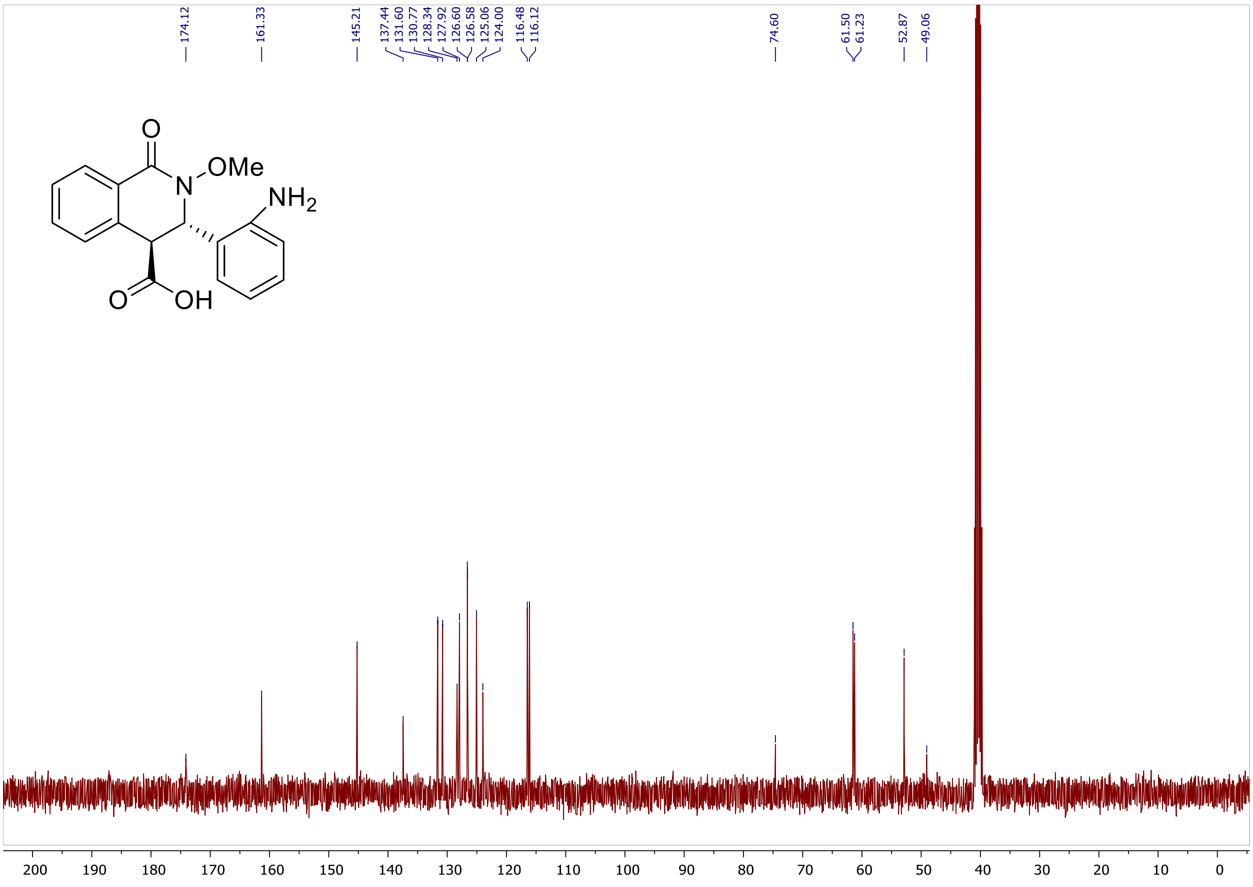

^{1}H- and ^{13}C-spectra of compound 8

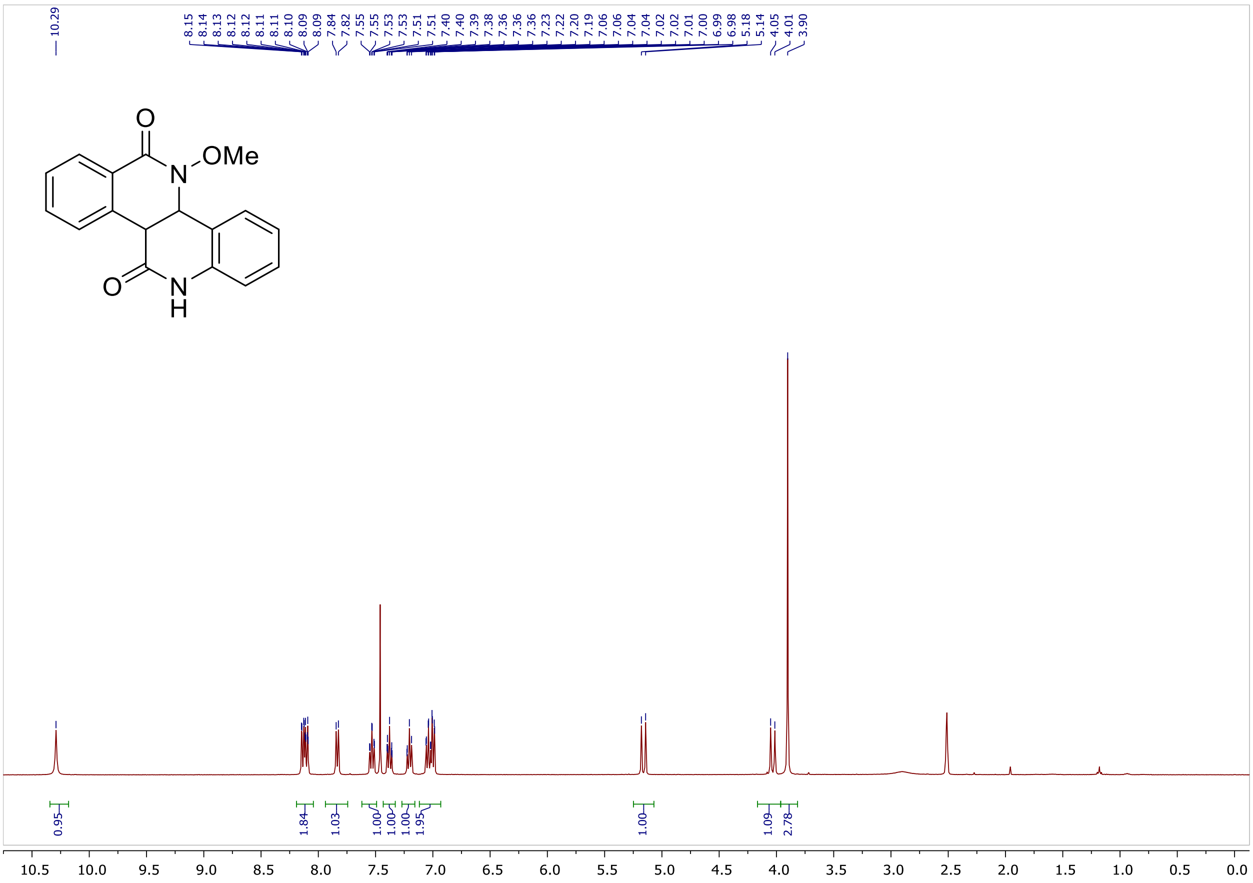

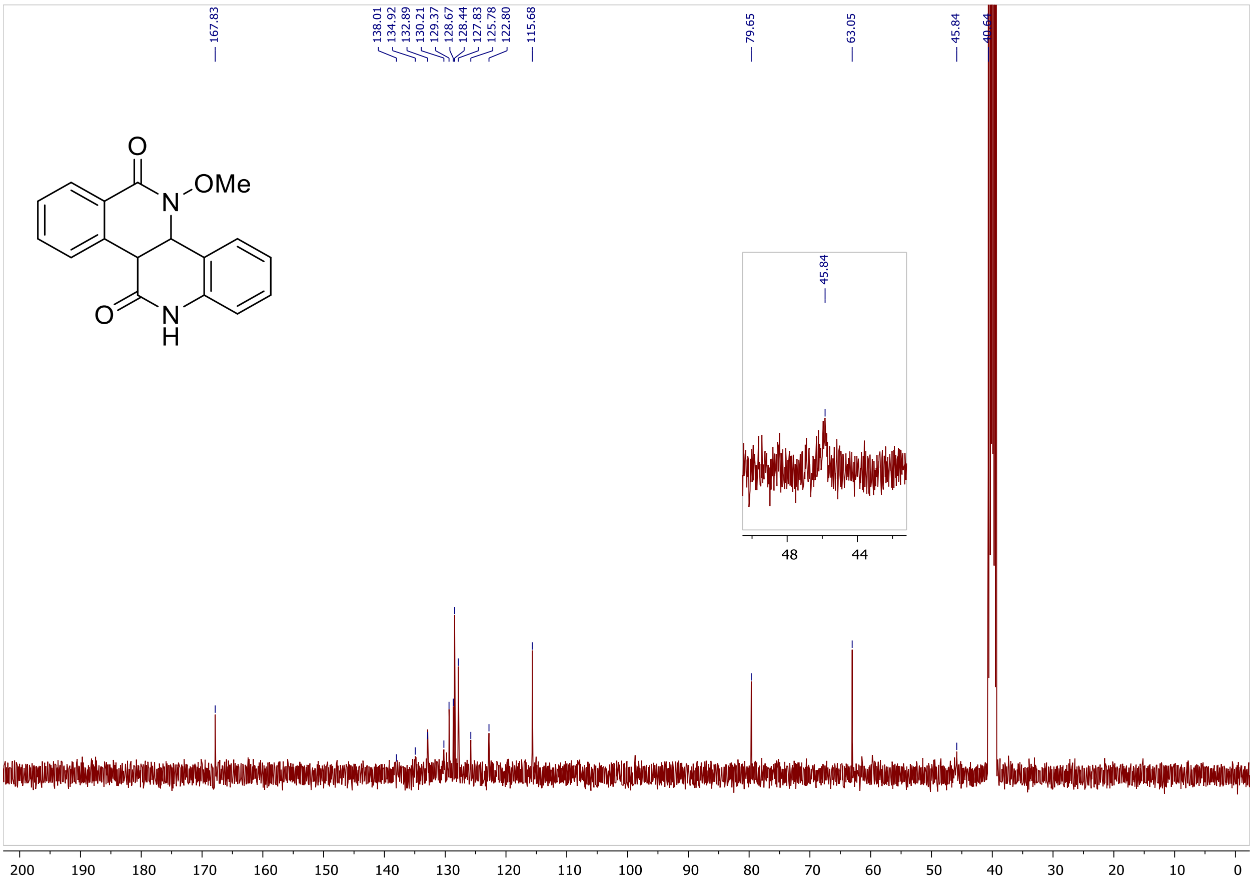

^{1}H- and ^{13}C-spectra of compound 9

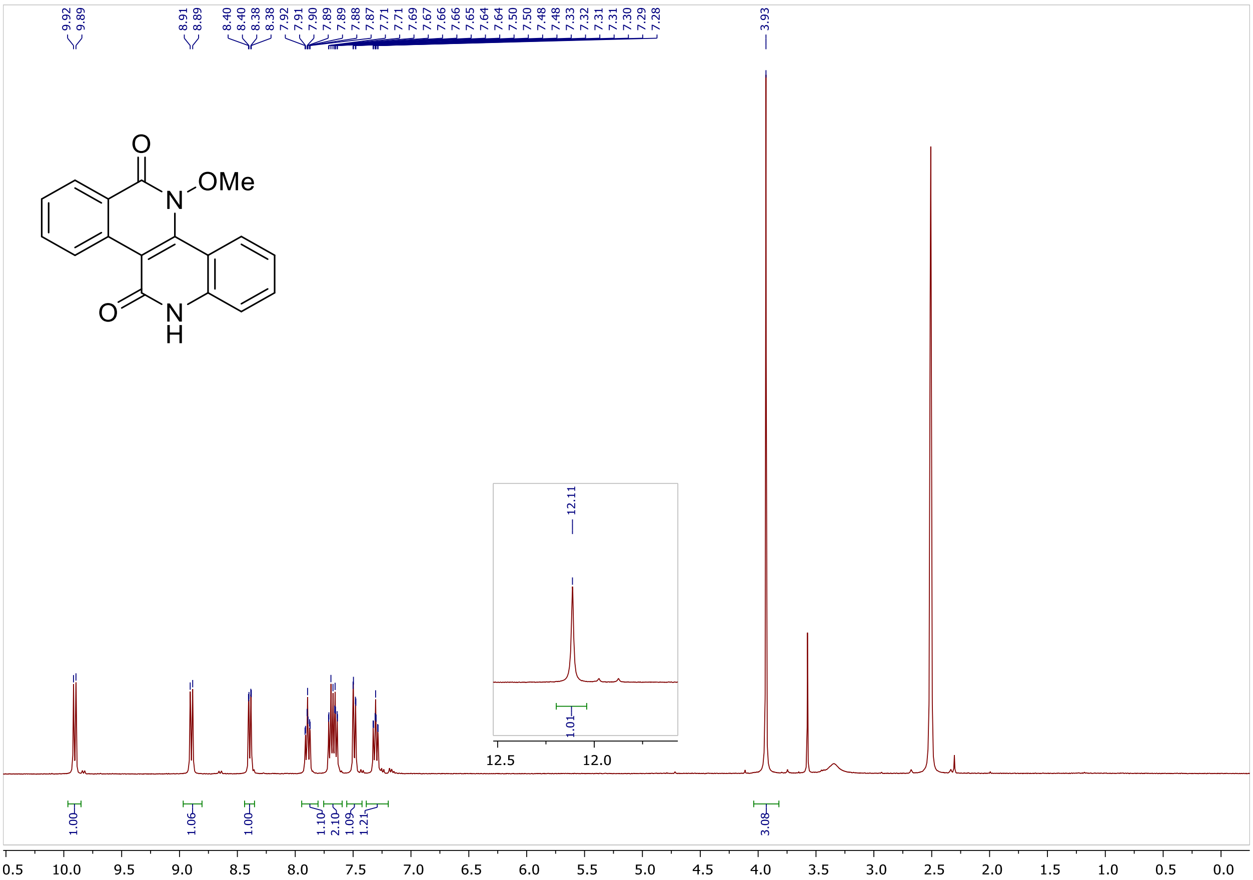

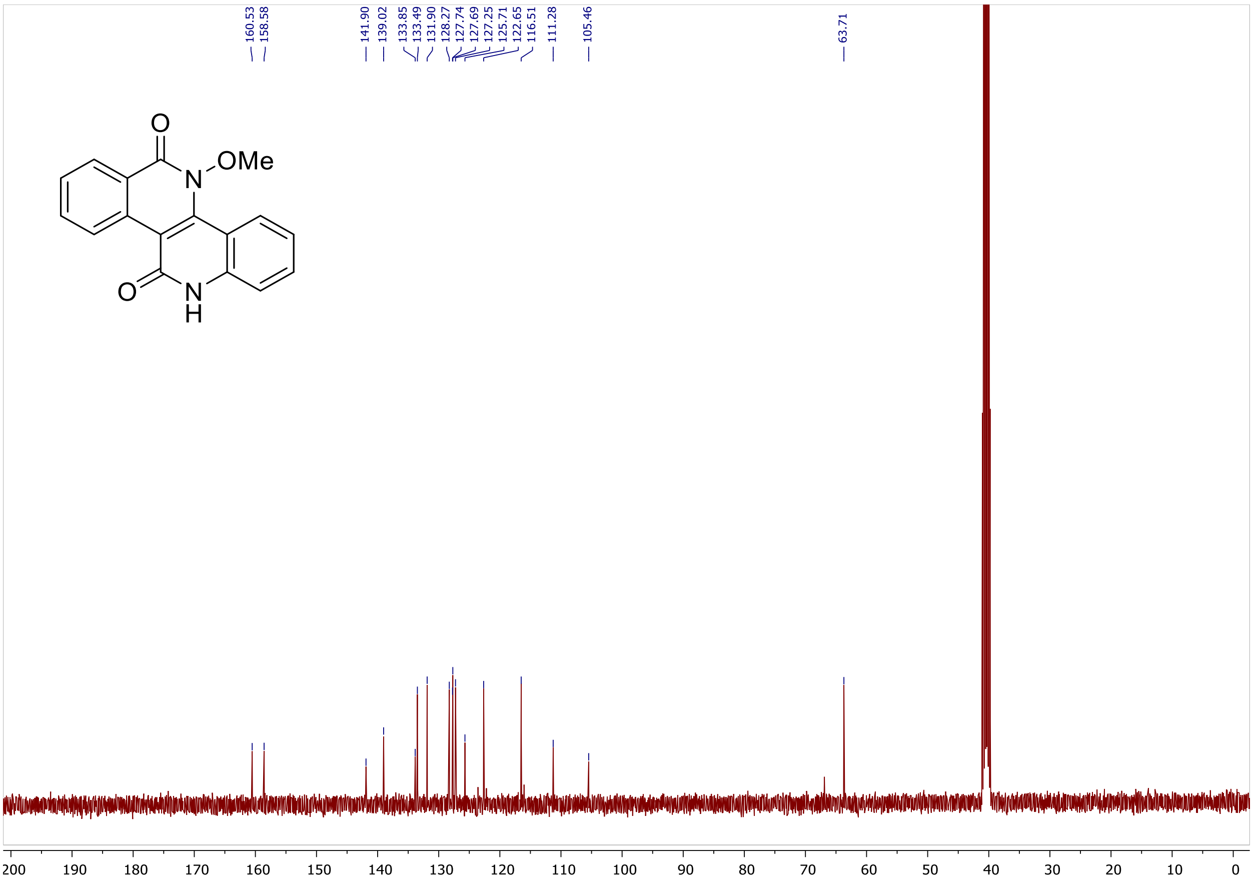

^{1}H- and ^{13}C-spectra of compound 2

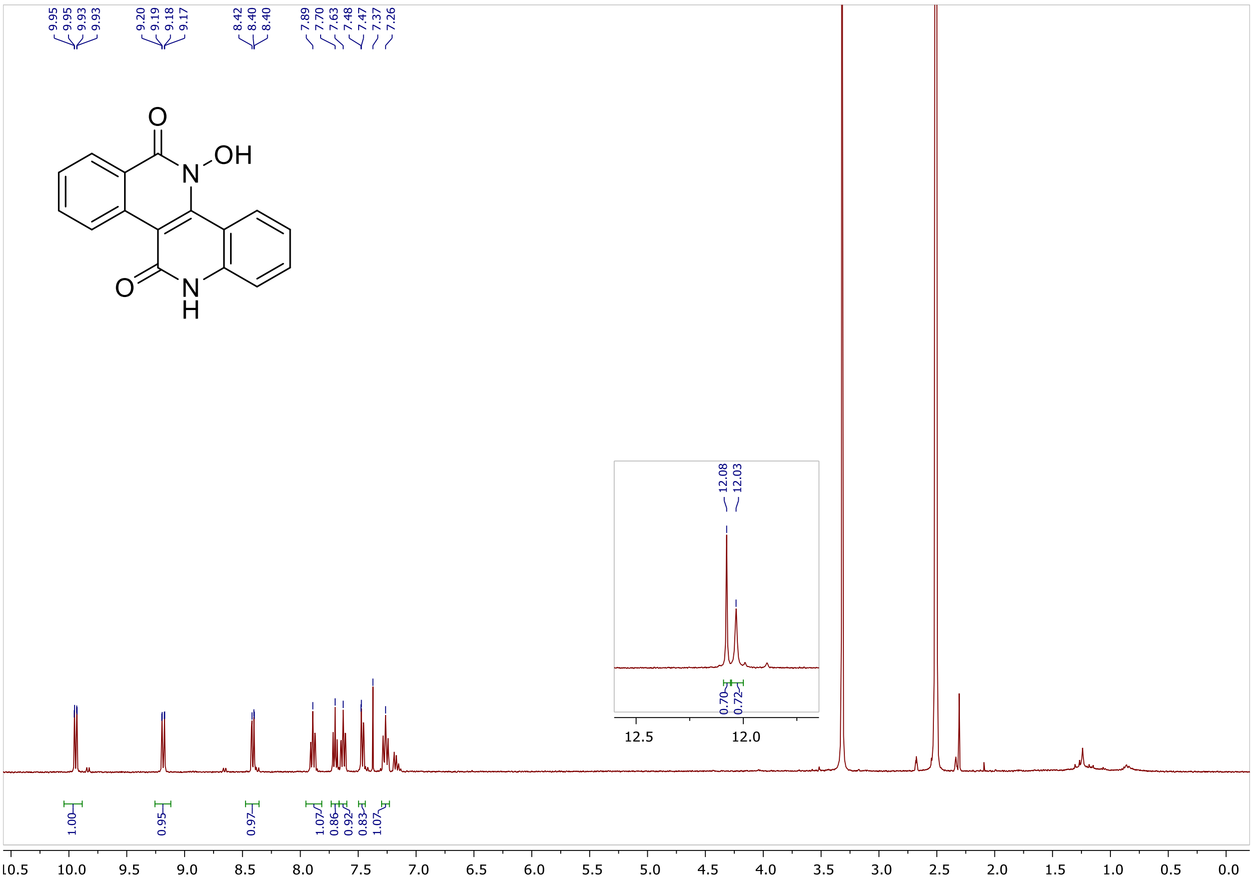

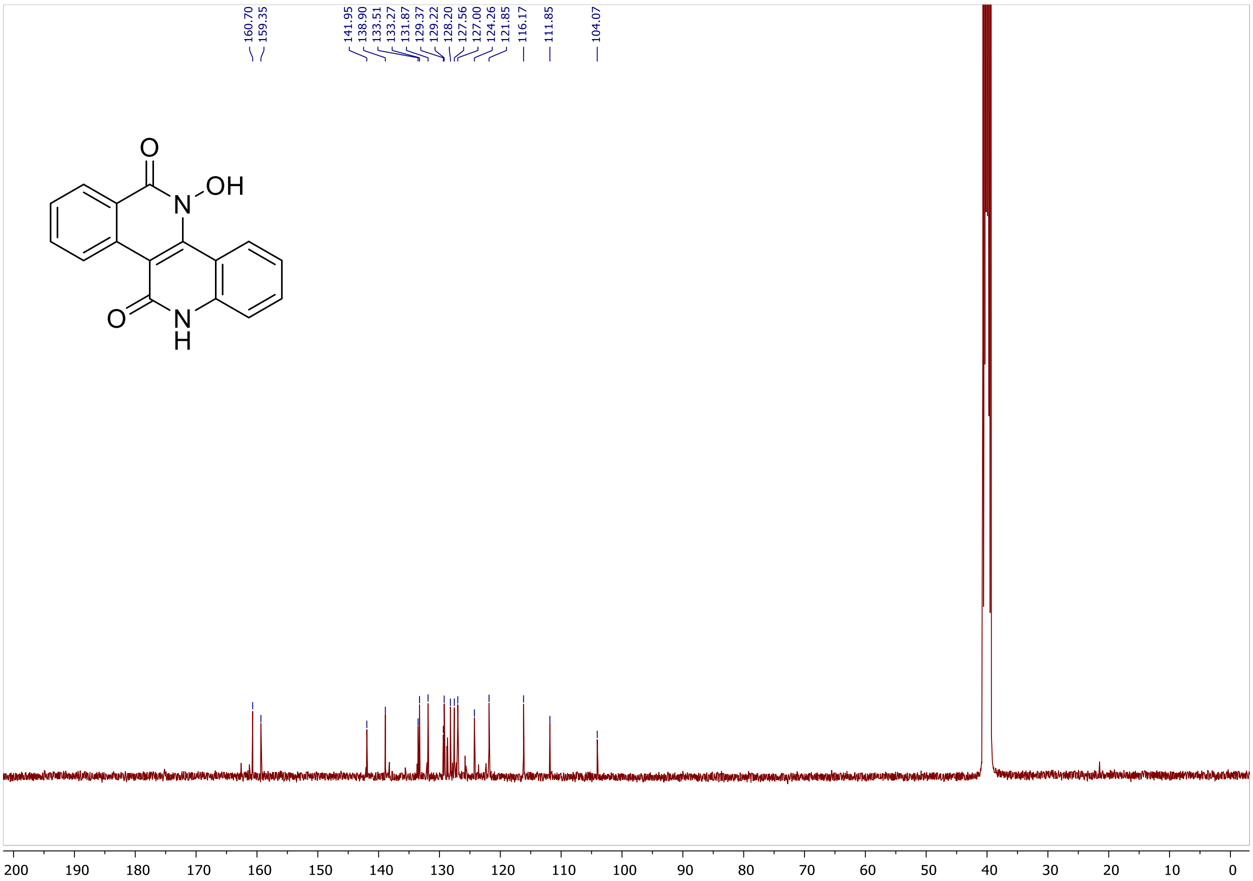

^{1}H- and ^{13}C-spectra of compound 10

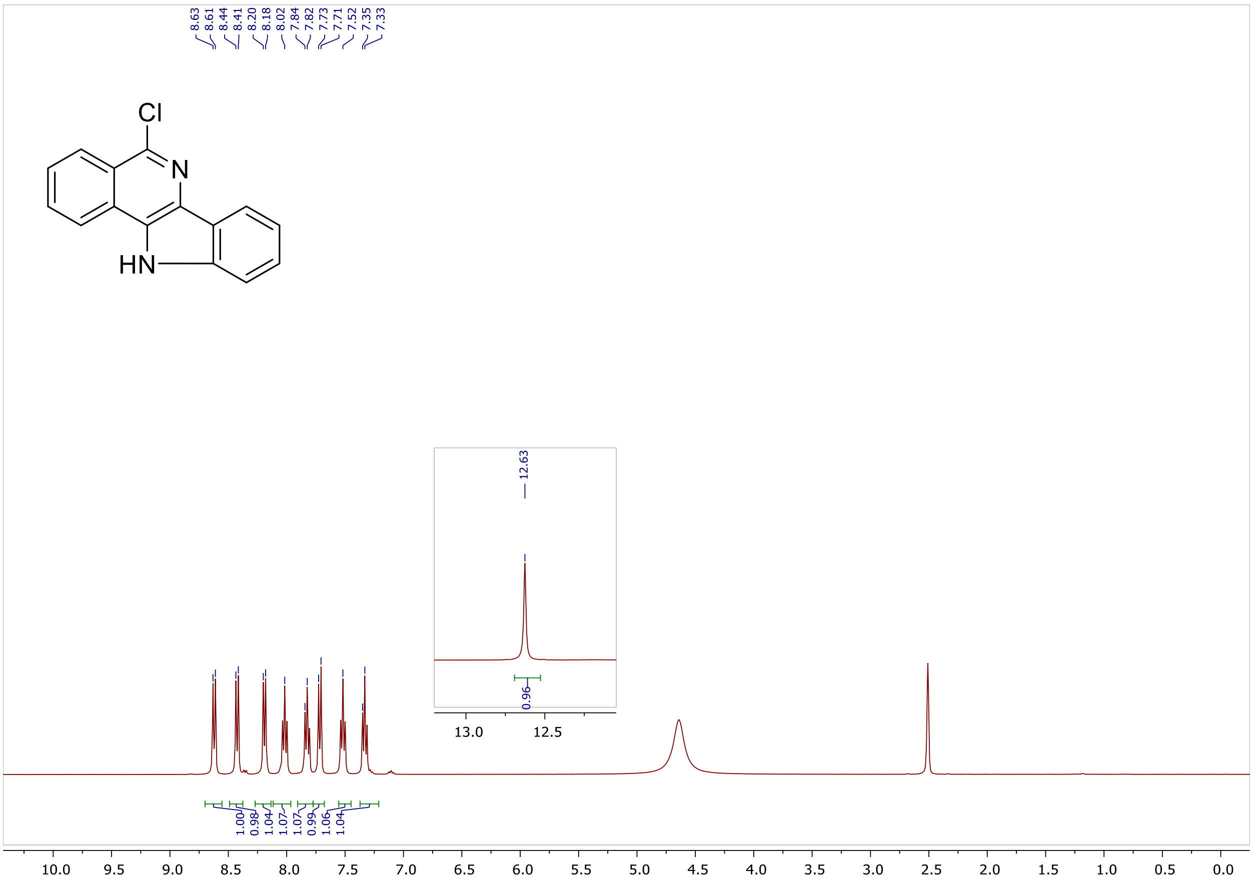

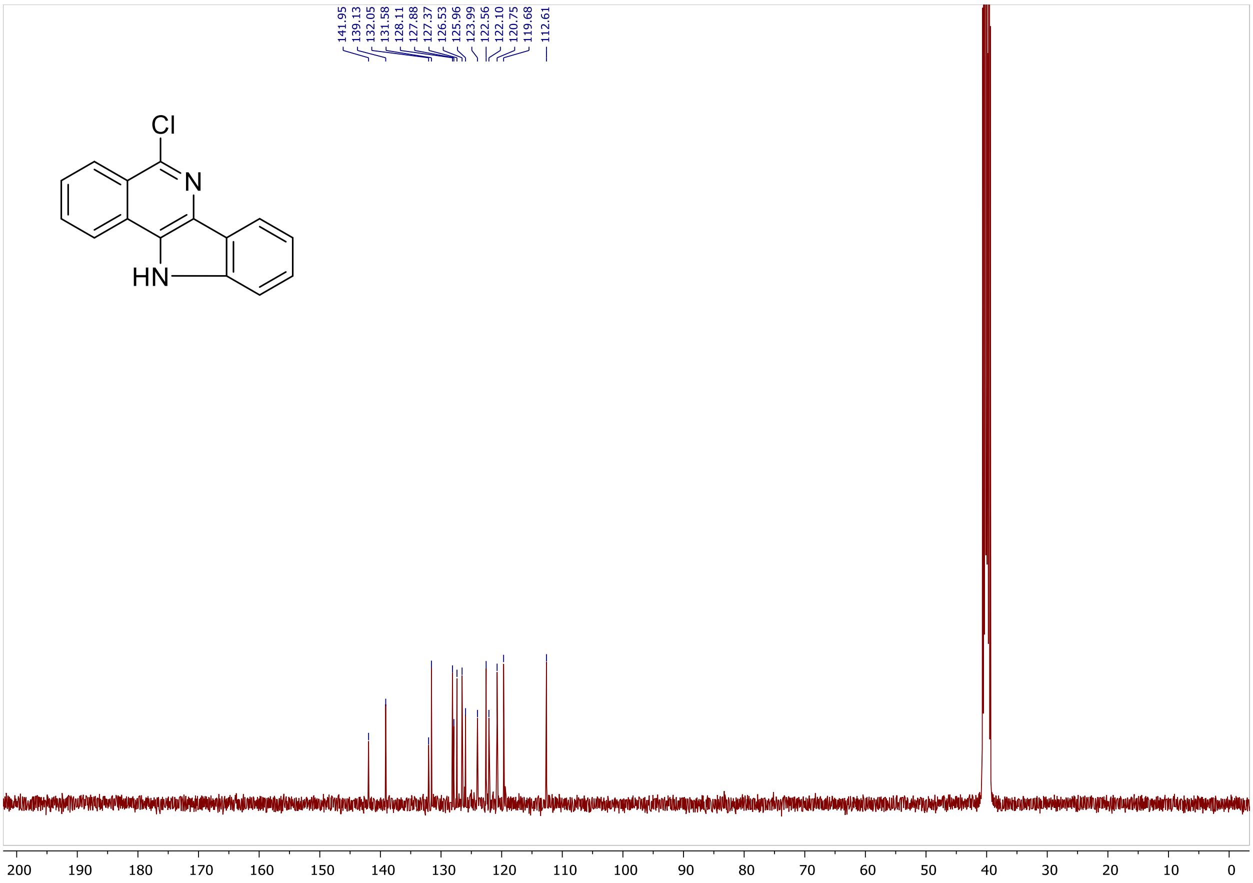

^{1}H- and ^{13}C-spectra of compound 11

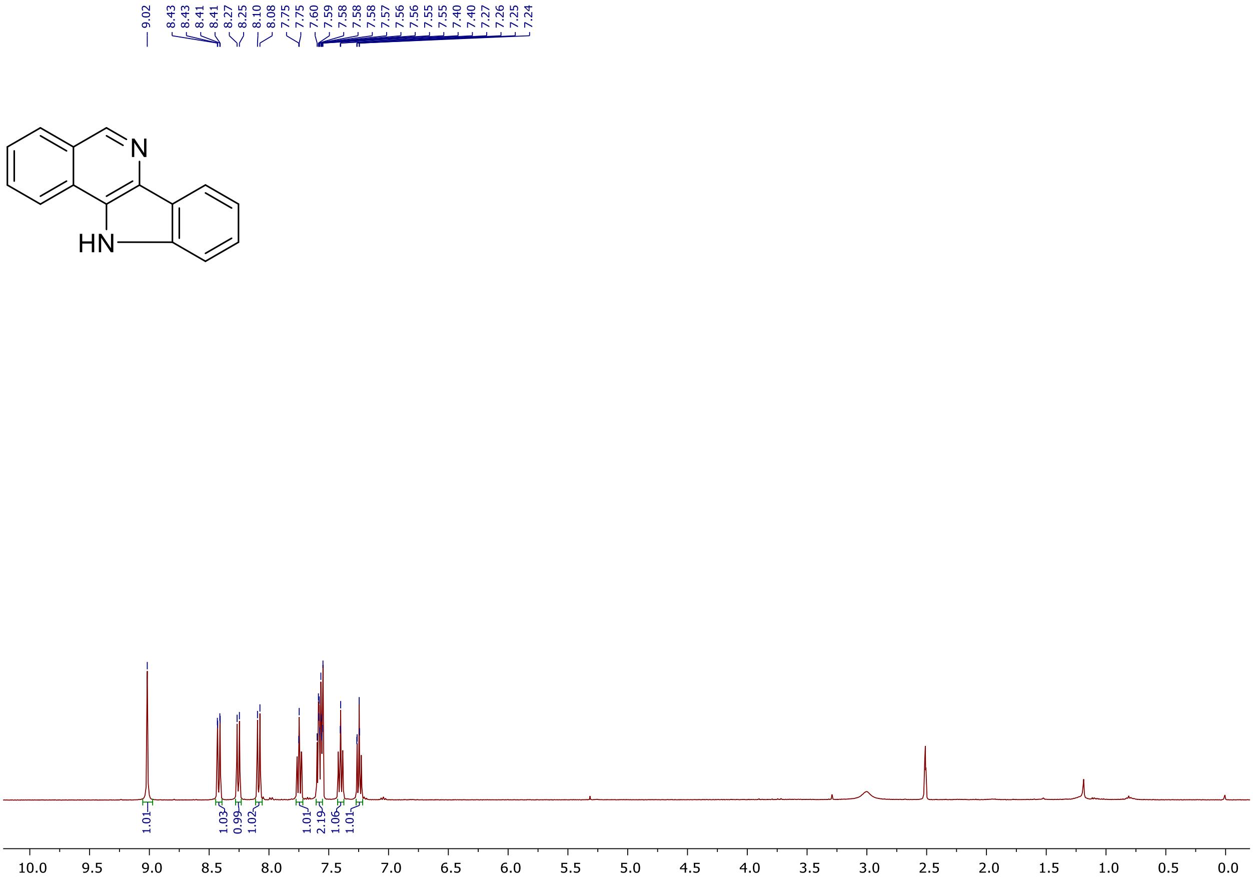

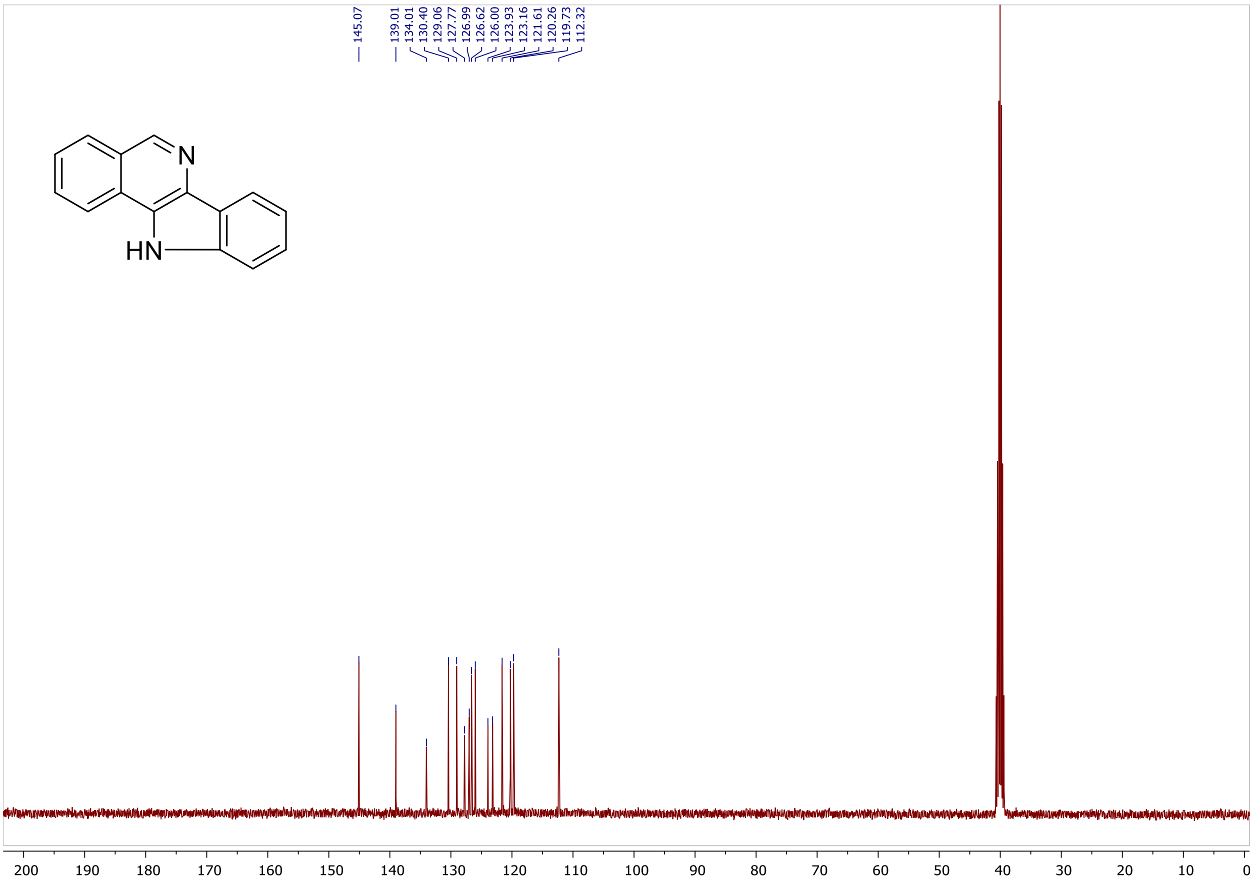

4. References

1. Dolomanov, O. V.; Bourhis, L. J.; Gildea, R. J.; Howard, J. A. K.; Puschmann, H., OLEX2: a complete structure solution, refinement and analysis program. *J. Appl. Crystallogr.* **2009,** 42, (2), 339-341, DOI: 10.1107/s0021889808042726.

2. Sheldrick, G. M., SHELXT - integrated space-group and crystal-structure determination. *Acta Crystallogr. A* **2015,** 71, (Pt 1), 3-8, DOI: 10.1107/S2053273314026370.

3. Sheldrick, G. M., Crystal structure refinement with SHELXL. *Acta Crystallogr. C* **2015,** 71, (Pt 1), 3-8, DOI: 10.1107/S2053229614024218.
